# How do miRNAs mediate translational repression?

**DOI:** 10.1186/1758-907X-1-11

**Published:** 2010-05-07

**Authors:** Shuo Gu, Mark A Kay

**Affiliations:** 1 Departments of Pediatrics and Genetics, Stanford University, Stanford, CA 94305, USA

## Abstract

Micro(mi)RNAs regulate gene expression by what are believed to be related but separate mechanistic processes. The relative contribution that each process plays, their mechanistic overlap, and the degree by which they regulate complex genetic networks is still being unraveled. One process by which miRNAs inhibit gene expression occurs through translational repression. In recent years, there has been a plethora of studies published, which have resulted in various molecular models of how miRNAs impair translation. At first evaluation, it appears that these models are quite different and incompatible with one another. In this paper, we focus on possible explanations for the various interpretations of these data sets, and provide a model that we believe is consistent with many of the observations published to date.

## Background

Since their discovery 16 years ago, the prominent role that micro(mi)RNAs play as key post-transcriptional regulators of genetic networks is becoming more apparent. Although significant progress has been made in understanding the biogenesis and biological function of miRNAs [1], the mechanism of how miRNAs inhibit a particular mRNA is still unclear. Single-stranded mature miRNAs associate with Argonaute (Ago) proteins to form the core of a multicomponent gene regulatory complex named the RNA-induced silencing complex (RISC). Guided by the sequence complementarity between the small RNA and the target mRNA, miRNA-RISC-mediated gene inhibition is commonly divided into three processes: (i) site-specific cleavage, (ii) enhanced mRNA degradation and (iii) translational inhibition. The first process is restricted to miRNAs with a perfect or near-perfect match to the target RNA; this is commonly referred to as RNA interference (RNAi), and in mammals it is carried out exclusively by Ago2. By contrast, the other two processes are more commonly associated with mismatched miRNA/target sequences, which is the most likely scenario in mammals. The combination of these two processes is commonly referred to as non-cleavage repression, and can be carried out by any of the four mammalian Ago proteins [2]. Interestingly, recent genetic [3] and biochemical [4] studies have established that plant miRNAs also induce translational repression on their target mRNA, despite the near-perfect complementarity between the miRNA and target sequences, indicating that non-cleaving repression may be the default conserved function of miRNAs in these two kingdoms. It is not entirely clear if enhanced mRNA degradation and translational inhibitory events are mutually exclusive or perhaps more inter-related. Recent evidence suggests that the two processes are fundamentally independent but may overlap in some situations. Interestingly, in the presence of miRNAs, some target mRNAs can be exclusively repressed by non-cleavage degradation or translational inhibition, but most repression probably occurs by some combination of the two processes (reviewed in [5]). Some studies [6,7] using a combination of genome-wide microarray and proteomic approaches demonstrated that the majority of miRNA-induced gene repression can be explained by a reduction in targeted mRNA levels. Nonetheless, because miRNA-mediated translational repression is well documented in numerous studies across many species and two kingdoms, it is a functionally operative process. However, it is fair to say that the quantitative contribution of miRNA-mediated translational repression in global post-transcriptional gene regulation is not resolved.

We focus this commentary on the mechanism(s) of how miRNAs inhibit mRNAs at the translational level. This is of interest because there have been a number of models based on fundamentally strong studies that nonetheless appear to be contradictory. As a result, data-driven examples suggest that the key regulatory blocks occur at various steps along the translational process. The rationale behind each model has been reviewed [5,8-11]. We point out the limitations of the various experimental approaches, and provide a model for translational repression that takes into account the various disparate results.

## Translational Repression: Why so many mechanisms?

Translational inhibition is established when the decrease in protein product is greater than the observed decrease in mRNA. Broadly divided, translation can be separated into three steps: ribosome initiation, elongation and termination. The primary method to establish the step at which translation is blocked is by measuring the location of an mRNA across a polysomal sucrose gradient. Early evidence in *C. elegans *[12,13], and more recent studies in mammalian cells [8,14-16] demonstrated that miRNA-repressed mRNAs maintained the same mRNA distribution pattern across poly-ribosomes (the polysomal profile) compared with non-repressed mRNAs. These results led to models of post-initiation inhibition of mRNA translation, including cotranslation peptide degradation [16], increased premature termination (ribosomal drop-off) [15], and impaired elongation [14]. By contrast, other groups observed that repressed mRNA specifically shifted to the lighter portion of the sedimentation velocity gradient, indicating less efficient ribosomal loading and thus miRNA-mediated repression at the translation initiation step [17-21].

How could such straightforward experiments generate such contrasting results? Although some of the differences may be due to relatively trivial variation in experimental procedures, other possibilities may be related to experimental oversights. For example, miRNAs induce target mRNA destruction in addition to translational repression [1]. The relative contribution of each of these inhibitory events will vary between targeted mRNAs. In some of the studies, the integrity of 'repressed' mRNAs was not carefully checked. Thus, the shifted reporter mRNA signals found during gradient sedimentation might represent partially degraded mRNAs, which dissociates with the translational machinery. In addition, miRNAs generally induce a smaller degree of repression (around two to three times) compared with site-specific RNAi-based cleavage. Whether all targeted mRNAs are partially repressed or a smaller population is fully repressed is an important distinction, which is still not resolved. Once this is established, it will have significant implications for the interpretation of a number of different mechanistic studies.

Another effort to distinguish initiation and post-initiation inhibition relied on testing whether internal ribosome entry sequence (IRES)-containing mRNAs were resistant to miRNA-mediated repression [22]. Like the polysomal profile experiments, contradictory results were reported by different groups. Some studies [15,23] demonstrated that IRES-initiated translation was still subject to miRNA-mediated repression, therefore excluding eukaryotic translation initiation factor 4E-cap recognition as a potential target for miRNA function. Other studies [17,24] concluded that miRNAs inhibit target mRNA translation at the initiation step. It is important to note that the IRES-initiated translation is generally much less efficient than that of m^7^G-capped mRNAs (1000 times in some cases). This inefficiency may alter translational kinetics, which in turn would have an affect on miRNA-mediated mRNA translational repression [25]. Finally, the host protein requirements for IRES mediated translation initiation vary depending on the biological origin of the IRES sequence. Thus, it is unclear if an IRES-mRNA is a valid substitute to study 5' cap-mRNA miRNA-mediated repressive states.

A third line of studies examined reconstituted cell-free extracts derived from rabbit reticulocytes [26], *Drosophila *[27] and mammalian cell lines [21,28], which support miRNA-mediated translational inhibition. These studies showed that the classic m^7^G 5' cap was required for repression, suggesting that miRNA-mediated translational inhibition occurred at the initiation step. Furthermore, in the study of Mathonnet *et al *[21], miRNA-mediated inhibition was alleviated by providing an excess of the eIF4F complex (eIF4E plus eIF4G) in the cell extracts, further suggesting the initiation step as the primary point of inhibition. However, the poor translational efficiency of reporter mRNAs in cell extracts, and the uncertainty about the effective concentration and bioavailability of other cellular factors that might be important in the process is an inherent weakness in such studies. In addition, because multiple mechanisms are operative, it is questionable how these reconstituted systems recapitulate the physiological events *in vivo*, given the fact that certain *in vitro *systems require overexpression of protein factors involved in the miRNA pathway [28].

Another set of important studies focused on novel methods to study miRNA/target and mRNA/protein interactions. For example, tethering Ago proteins, the core of the miRNA effector complex (RISC), to a 3' untranslated reading frame without an associated miRNA still induced translational repression [29]. Therefore, it became relevant to further study how the Agos or Ago-associated proteins interact with translational machinery. Indeed, Kiriakidou *et al*. [30] reported that mammalian Ago2 binds to the m^7^G-cap directly. This supports the notion that Ago2 and eIF4E compete for association with the target mRNA cap structure and thus prevent effective translation initiation. This model is consistent with the results obtained from cell reconstitution studies, and has been widely cited as the key evidence to support the idea that miRNAs block translational initiation. However, structural studies were not consistent with the proposed interaction between the cap structure and the Ago2 MC domain, previously believed to be directly responsible for the interaction [31]. In addition, other studies [2,9] provided an alternative explanation for the role of the Ago MC domain, when it was shown that Ago2 did not specifically bind to the 5' cap. Instead, the mutation of two key amino acids in the MC domain, which were intended to disrupt the cap-Ago2 binding actually abolished the association of Ago2 and GW182, a cellular factor known to be crucial for miRNA-mediated repression (see below). Of note, a recent study proposed an allosterically regulated 5' cap binding motif within the Ago MID domain, which upon the association of the Ago protein and guiding strand/target of RNA duplexes, inhibits translation at the initiation step [32].

Other studies provide a direct role for eIF6, a protein factor that prevents the association of ribosomal subunits [19], as a key mediator in miRNA-mediated translational repression. Based on the observations that eIF6 was co-immunoprecipitated with miRISC, and that knockdown of eIF6 resulted in the alleviation of miRNA-mediated repression in *C. elegans*, the authors concluded that miRNAs inhibit translation by preventing the joining of 60S and 40S subunits to make an 80S ribosome. This model was nicely supported by an acellular reconstitution study [33] demonstrating that miRNA-repressed mRNAs were enriched in 40S but not 60S ribosomes. However, these results were not reproduced in *Drosophila *S2 cells [9]. In addition, the involvement of eIF6 in ribosome biogenesis [34,35] further weakens the original conclusion and indicates the effect of eIF6 on miRNA-mediated repression is indirect.

## The role of GW182 in miRNA-mediated Repression

Several recent studies indicate that GW182 family proteins interact directly with Agos, and are required for miRNA-mediated gene silencing in *C. elegans*, *Drosophila *and mammalian cells [9,36]. As a P-body component, GW182 was originally linked to the miRNA pathway by various genetic screening and biochemical studies [9,36]. The observation that GW182 mediated gene silencing in a pattern similar to that of miRNA, when artificially tethered to an mRNA reporter without any miRNA target sequences, clearly demonstrates that GW182 can function independently from and perhaps downstream of Agos [9]. GW182 was originally believed to be responsible for miRNA-mediated non-cleavage degradation of target mRNA by specifically bridging RISC and deadenylase CCR4-NOT, which caused deadenylation followed by uncapping of target mRNAs [9]. Recent evidence suggested that miRNA-mediated translational repression also requires GW182 [9,20]. Therefore, the interactions between GW182 and the translational machinery seem to be moving towards providing a more accurate depiction of the steps involved in translational silencing. Indeed, recent studies by Fabian *et al*. [21] and Zekri *et al*. [37], despite the discrepancies in details, both identified poly-A binding protein (PABP) as the factor specifically associated with GW182 in *Drosophila *and mammalian cells. These findings suggest that GW182 may disrupt the association between eIF4G and PABP, which is required for efficient translation initiation. However, the observation that mRNAs without poly-A tails are still subject to miRNA-mediated translational repression [9,38] strongly suggests that there are additional translational repression factors involved besides the eIF4G-PABP association.

A recent report from Isawaki *et al *[39] provides more clues as to why there may be multiple operative mechanisms to induce miRNA-mediated translational repression. *Drosophila *Ago1 and Ago2 were reported to mediate target gene repression through distinct mechanisms. Whereas Ago1 elicits deadenylation and in turn target mRNA degradation through GW182, Ago2 induces translational repression independent of GW182 by association with eIF4E, disrupting the eIF4E-eIF4G interaction, which is essential for efficient translation. Although these results may explain some of the discrepant results in the fly, it is still far from solving the issue in mammals. Mammalian argonaute proteins (Ago1 to Ago4) are structurally, phylogenetically and functionally similar to *Drosophila *Ago1, but not Ago2.

## Conclusion: Our model

**Figure 1:**
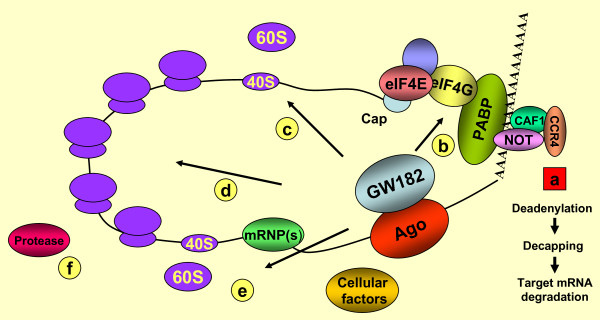
**Model for miRNA-mediated repression**. The interaction between GW182 and any of the Ago proteins is the first event of or occurs during micro(mi)RNA-target interaction. Several different pathways downstream of GW182 are possible. The specific pathway is probably determined by the composition of the RNA-induced silencing complex (RISC) and interaction with mRNA-ribonucleoprotein (mRNP) complex), microRNA-associated ribonucleoproteins (miRNPs), or cellular factors. **(a) **GW182 mediates deadenylation, followed by de-capping and mRNA degradation via NOT/CCR4/CAF1 complexes; this is the primary non-cleaving degradation pathway and considered separate from the translational repression pathway. **(b) **GW182 competes with eIF4G in association with poly-A binding protein (PABP), preventing the circularization required for efficient translation. **(c) **The 60S ribosome is prevented from joining with the 40S ribosome to form 80S ribosomes. Steps **(b) **and **(c) **represent initiation blocks. **(d) **Slowed or stalled ribosomes along the mRNA, representing a translation elongation block. **(e) **Premature translation termination and **(f) **co-translation degradation.

How can we reconcile the multiple proposed mechanisms with the finding that GW182 plays a key role in miRNA-mediated translational repression? It is possible that the interaction between GW182 and one of the Agos is the primary event leading to the activation of several pathways. A model is presented in Figure 1. In this model, Agos do not directly mediate repression, but rather function together with loaded miRNA guiding strands, which function as an adaptor to dock GW182 to target mRNAs. The N-terminal portion of GW182 specifically associates with the Ago protein and mediates gene repression through an interaction between its C-terminal and the various downstream factors. The final type and level of repression is a sum of the results from multiple pathways. Which pathway(s) dominate for a specific mRNA will depend on the specific Ago protein, and on *cis *(such as components of mRNA-ribonucleoprotein (mRNP) complex) and *trans *factors. Supporting this idea, Kong *et al*. [40] reported that the mechanism of miRNA-mediated repression might depend on the promoter used for driving the target mRNA expression. These authors proposed that different promoters result in a distinct set of mRNPs on reporter mRNA, which in turn determine the type of translational repression mechanism. It will be of great interest to identify these other factors and establish their roles in regulating miRNA-mediated non-cleavage repression.

Returning to the model in Figure 1, it is likely that translational inhibition and enhanced mRNA degradation are uncoupled processes with the relative participation of each for a particular mRNA-miRNA defined by the differences in the protein-RNA interactions. For example, the relative degree of repression dictated by degradation (Figure 1, process (a)) versus translational inhibition (processes (b) to (f)) will be related to the proportion of specific complexes that form for a particular population of mRNAs. Understanding the rate-limiting processes in the formation of such complexes is likely to be the key to a better understanding of post-transcriptional gene regulation.

Nevertheless, it is likely that even within the process of translational inhibition, the mechanism is much more finely regulated than originally anticipated. The fact that there are many studies supporting translational inhibition at each of the three defined levels of translational inhibition (initiation, elongation and termination) suggest that during repression all three are coordinately stalled or slowed (see Figure 1, processed (b) to (f)). This single model is consistent with the finding that miRNA-mediated repressed mRNAs have the same average polysomal distribution compared with those that are not repressed. However, it is possible that a one or more process(es) (for example, Figure 1, process (b) to (f)) predominate for specific mRNA-RISC complexes. Together, this model takes into account the various differences reported by various investigators.

A wider question is: why are there multiple processes for miRNA-mediated gene silencing? Is there an advantage for some mRNAs to be slowed or stalled on polysomes so that their product levels can be quickly turned up or down? Conceivably this might allow more precise regulation compared with mRNA destruction. Multiple approaches including elucidating the molecular events will be contributory to our understanding of this complex set of regulatory processes that seems to be required for such precision in controlling gene expression.
